# Molecular pathways identified from single nucleotide polymorphisms demonstrate mechanistic differences in systemic lupus erythematosus patients of Asian and European ancestry

**DOI:** 10.1038/s41598-023-32569-6

**Published:** 2023-04-01

**Authors:** Katherine A. Owen, Kristy A. Bell, Andrew Price, Prathyusha Bachali, Hannah Ainsworth, Miranda C. Marion, Timothy D. Howard, Carl D. Langefeld, Nan Shen, Jinoos Yazdany, Maria Dall’era, Amrie C. Grammer, Peter E. Lipsky

**Affiliations:** 1 AMPEL BioSolutions LLC and the RILITE Research Institute, Charlottesville, VA 22902 USA; 2 Department of Biostatistics and Data Science, Center for Precision Medicine, Wake Forest School of Medicine, Winston-Salem, NC 27109 USA; 3 Department of Biochemistry, Center for Precision Medicine, Wake Forest School of Medicine, Winston-Salem, NC 27109 USA; 4 Shanghai Institute of Rheumatology, Renji Hospital, Shanghai Jiao Tong University School of Medicine, Shanghai, China; 5 University of California San Francisco, San Francisco, CA 94117 USA

**Keywords:** Immunogenetics, Gene regulatory networks, Systemic lupus erythematosus, Gene expression profiling, Computational biology and bioinformatics, Genetics, Immunology, Risk factors

## Abstract

Systemic lupus erythematosus (SLE) is a multi-organ autoimmune disorder with a prominent genetic component. Individuals of Asian-Ancestry (AsA) disproportionately experience more severe SLE compared to individuals of European-Ancestry (EA), including increased renal involvement and tissue damage. However, the mechanisms underlying elevated severity in the AsA population remain unclear. Here, we utilized available gene expression data and genotype data based on all non-HLA SNP associations in EA and AsA SLE patients detected using the Immunochip genotyping array. We identified 2778 ancestry-specific and 327 trans-ancestry SLE-risk polymorphisms. Genetic associations were examined using connectivity mapping and gene signatures based on predicted biological pathways and were used to interrogate gene expression datasets. SLE-associated pathways in AsA patients included elevated oxidative stress, altered metabolism and mitochondrial dysfunction, whereas SLE-associated pathways in EA patients included a robust interferon response (type I and II) related to enhanced cytosolic nucleic acid sensing and signaling. An independent dataset derived from summary genome-wide association data in an AsA cohort was interrogated and identified similar molecular pathways. Finally, gene expression data from AsA SLE patients corroborated the molecular pathways predicted by SNP associations. Identifying ancestry-related molecular pathways predicted by genetic SLE risk may help to disentangle the population differences in clinical severity that impact AsA and EA individuals with SLE.

## Introduction

Systemic lupus erythematosus (SLE) (OMIM:152700) is a complex autoimmune disease characterized by clinical and genetic heterogeneity. Individuals of East Asian ancestry (AsA) have a greater prevalence of renal involvement, infections and cardiovascular complications compared to individuals of European ancestry (EA)^1^. In particular, lupus nephritis and end stage renal disease (LN/ESRD) are severe complications of SLE that are more prevalent in patients of AsA ancestry than patients of EA ancestry^2–4^. Whereas some of this variation may be accounted for by confounding environmental and/or socioeconomic factors^5^, it is unclear why AsA ancestry remains associated with clinical severity and sub-phenotypes in SLE.

Immunochip-based and genome-wide association (GWA) studies have revealed important ancestry-specific and trans-ancestral risk associations predisposing to SLE^6–10^. Recent meta-analyses of European and Chinese GWAS data suggest that the greater disease burden evident in East Asian populations is at least partially a consequence of different risk variant frequencies^9^. Although these and other studies enable a better understanding of the genetic architecture of SLE, they tend to focus on only the most significant associations and linked genes, and thereby do not capture the totality of genetic variation. Critically, genetic analyses to date have been unable to provide a clear path toward novel therapeutic development. This shortcoming is of particular concern with respect to AsA patients, where the control of disease activity remains suboptimal^5^. Here, we undertook a bioinformatics-driven approach to identify a comprehensive list of ancestry-specific and shared SLE-associated genes, using eQTL mapping, the identification of functional variants in coding regions and variants impacting transcription factor binding site occupancy, as well as SNP-gene proximity. Together, this approach identified 3105 potential SLE-associated genes in one or more ancestral groups (1349 EA, 1429 AsA and 327 trans-ancestral). Connectivity mapping and network analysis were used to identify ancestry-enriched biological pathways and inform ancestry-specific pharmacological targets. This trans-ancestral analysis strategy not only identified additional SLE-associated molecular pathways but, due to the underlying differences between AsA and EA in risk-allele frequencies, may enable a deeper understanding of the differences in the prevalence of SLE risk, severity, and clinical phenotypes. Such an understanding may motivate population-specific clinical trials and interventions.

## Results

### Identification of ancestry-dependent and independent non-HLA SLE-associated variants and downstream target genes

**Figure 1:**
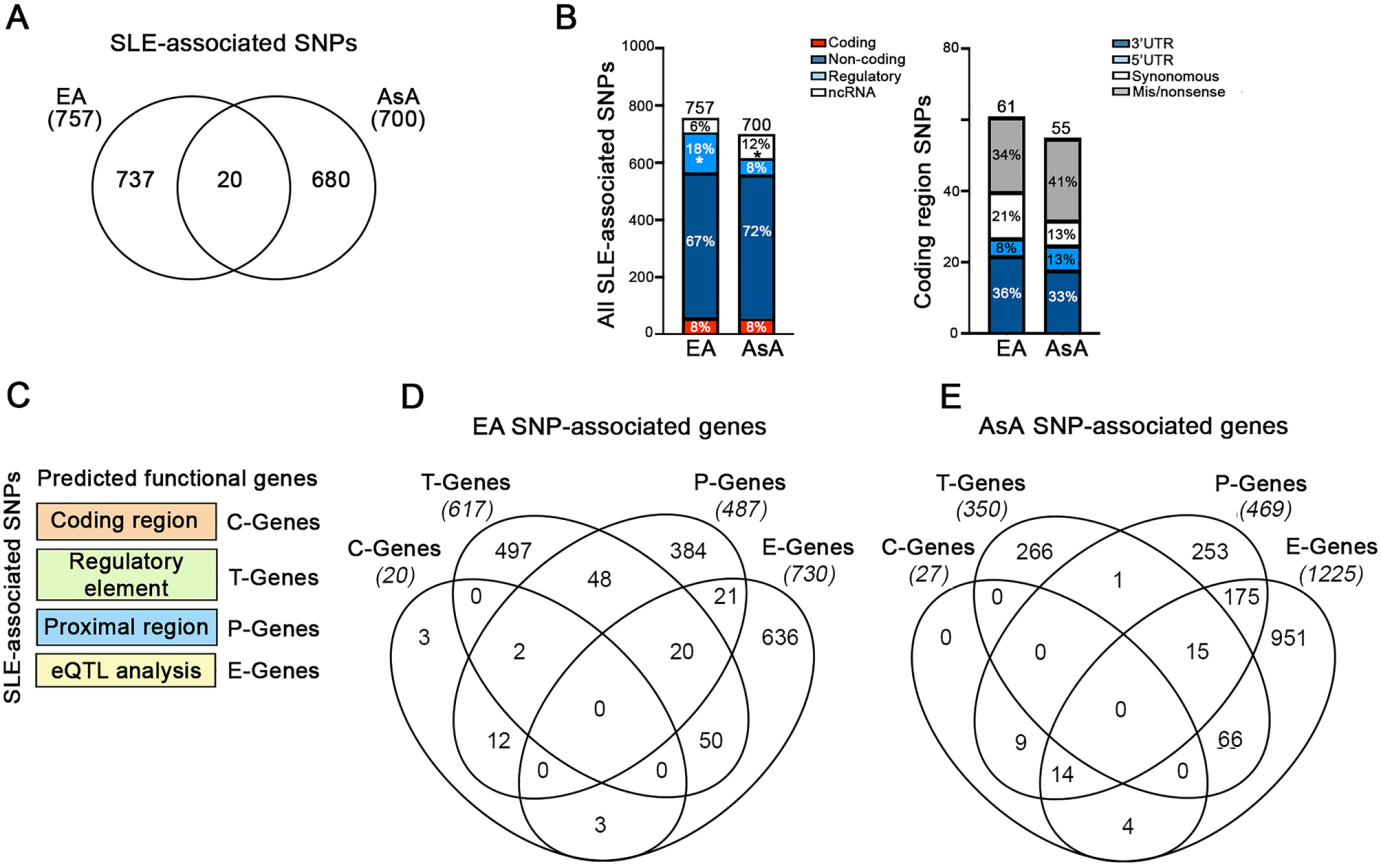
Mapping the functional genes associated with SLE-Immunochip SNPs. (**A**) Venn diagram depicting the ancestral overlap of all SLE-associated Immunochip SNPs. (**B**) Distribution of genomic functional categories for all EA and AsA non-HLA associated SLE SNPs. Genomic category comparisons between ancestral groups were performed using a 2-proportion* z* test. P values were 2-tailed, and asterisks indicate a significance threshold of p < 0.05. (**C**) Functional SNP-associated genes are derived from 4 sources, including eQTL analysis (E-Genes), regulatory regions (T-Genes), coding regions (C-Genes) and proximal gene-SNP annotation (P-Genes). (**D**, **E**) Venn diagrams showing the overlap of all EA (**D**) and AsA (**E**) associated E-, T-, C- and P-Genes.

Despite the success achieved by GWAS in mapping polygenic disease risk loci in SLE, the biological implications of the majority of identified variants has remained unknown. To gain a broader view of how inherited genetic variation impacts disease risk, we took the global approach of integrating SNPs with a range of association significance to generate a cohort of predicted genes that could ultimately be pruned and mapped to functional pathways for analysis. Immunochip-based association analyses have identified 700 single-nucleotide polymorphisms (SNPs) reported as significantly associated with SLE in patients of East Asian (AsA) ancestry^6^ and 757 SNPs associated with disease in European (EA) populations (Fig. 1A)^8^. Twenty SNP associations (< 1.5%) were shared between ancestries. In both ancestries, approximately 70% of SNPs were found in non-coding regions (intergenic and intronic), and 8% of SNPs were in coding regions (3′UTRs, 5′UTRs, synonymous and non-synonymous) (Fig. 1B and Supplemental Table S1). AsA populations had a significantly higher percentage of SLE-associated SNPs in non-coding (nc)RNAs (lncRNA and miRNA), whereas EA populations had more SLE-associated SNPs located within regulatory regions, including enhancers, promoters, open chromatin, and transcription factor binding sites (Fig. 1B).

We used a bioinformatics-based approach to identify the most plausible genes affected by the SLE-SNP association. As previously described^11,12^, we first determined whether there was evidence that the SNP was an expression quantitative trait loci (eQTL) using the GTEx database (version 8) and the Blood eQTL browser^13^. 226 EA- and 405 AsA- SLE-associated eQTLs linked to 730 and 1225 expression genes (E-Genes), respectively (Fig. 1C–E and Supplemental Table S2). eQTLs were identified for nearly 60% of the total number of AsA Immunochip SNPs, compared to 29% of SLE-associated SNPs in the EA data (Supplemental Fig. S1). Whereas the number of AsA eQTLs was higher across all genomic categories (coding, non-coding and regulatory regions as well as ncRNAs), the proportion of AsA SLE-associated SNPs linked to ncRNAs (51%; 42/82) was nearly three times higher than that observed in EA populations (18%; 8/44) (Supplemental Fig. S1 and Supplemental Table S1). Next, we sought to identify SNPs within distal and *cis* regulatory elements (e.g., enhancers and promoters), using the computational tools GeneHancer and HACER (Human ACtive Enhancers to interpret Regulatory variants), both of which connect regulatory SNPs with downstream target genes (T-Genes)^14,15^. Together, GeneHancer and HACER identified 105 SLE-associated SNPs (59 EA, 36 AsA) overlapping distal regulatory elements or promoters predicted to impact the expression of 964 T-Genes (617 EA, 350 AsA) (Fig. 1C–E and Supplemental Table S2). For variants located in coding regions, 44 SNPs (21 EA, 23 AsA) were associated with either non-synonymous or nonsense changes in 47 genes (C-Genes; 20 EA, 27 AsA) (Supplemental Table S2). The remaining SNPs that were not linked to E-, T- or C-Genes were assigned to the closest proximal gene (P-Gene), identifying an additional 956 P-Genes (487 EA, 469 AsA) (Supplemental Table S2).

**Figure 2:**
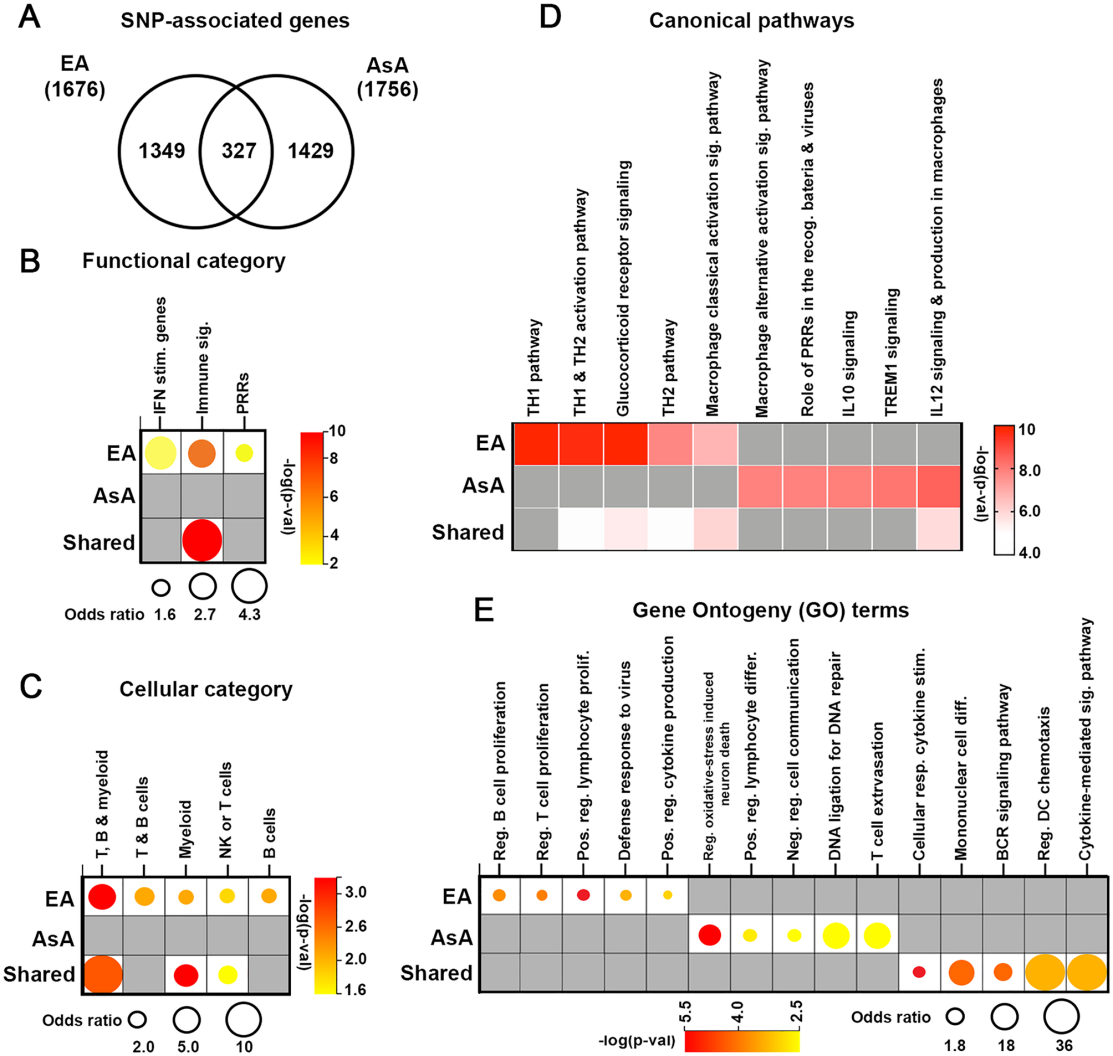
Functional characterization of SNP-associated genes. (**A**) Venn diagram depicting the overlap between all EA- and AsA-SNP associated genes. (**B**, **C**) Bubble plots depict ancestry-dependent and independent SNP-associated genes analyzed to determine enrichment using functional definitions from the BIG-C (Biologically Informed Gene Clustering) annotation library and I-Scope for hematopoietic cell enrichment. Enrichment was defined as any category with an odds ratio (OR) > 1 and a − log (p-value) > 1.33. (**D**) Heatmap (generated by GraphPad Prism 8.3; www.graphpad.com) visualization of the top five significant IPA canonical pathways and (**E**) bubble plot showing gene ontogeny (GO) terms for each gene list organized by ancestry. Top pathways with OR > 1 and − log (p-value) > 1.33 are listed.

Overlapping EA and AsA SNP-linked E-, T-, C- and P-Genes are depicted in Fig. 1D,E, respectively. No genes were shared within all four groups within either ancestry, and we observed limited commonality between T-, P- and E-Genes, with only 20 genes shared among the three groups in EA and 15 genes shared in AsA. It is notable that of the total of 3,432 SNP-linked genes, < 10% (327) overlapped between AsA and EA lupus cohorts (Fig. 2A).

### Characterization of gene signatures

We next completed a series of bioinformatic analyses to determine the overall biological function of the 1349 genes associated with SLE in EA and 1429 genes associated with SLE in AsA, as well as the 327 SLE-associated genes common to both ancestries. Analysis of genes linked through EA revealed enrichment in processes related primarily to adaptive immune function, including the functional category for interferon stimulated genes, canonical pathways for *TH1 and TH2 activation pathway* and *Macrophage classical activation signaling pathway*, and GO terms for the Regulation of B cell proliferation (GO:0030888) and the Regulation of T cell proliferation (GO:0042129). Genes linked to SNPs associated with SLE in the AsA cohort were enriched in categories related to pathogen-influenced signaling, such as *Role of PRRs in the recognition of bacteria and viruses,* and the Positive regulation of lymphocyte differentiation (GO:0045621), as well as those representing more diverse biological functions, such as Regulation of oxidative stress-induced neuron death (GO:1903203) and DNA ligation involved in DNA repair (0051103). Shared genes were distributed in a range of adaptive and innate immune gene categories (Fig. 2B,D,E).

In addition, EA- and AsA-derived gene sets were examined using a clustering program that detects immune and inflammatory cell type signatures within large gene lists to identify dominant immune cell populations driving disease pathology within each ancestry^16^. Consistent with our pathway analysis, EA exhibited strong enrichment in cellular categories for myeloid, T, and B cells, whereas SLE-associated genes in AsA were not enriched in any cellular category (Fig. 2C). Independent analysis of shared genes revealed enrichment in the T, B and myeloid, and the NK or T cell categories. Finally, parallel analyses examining P-Genes separately from E-, T-, and C-Genes were conducted to assess the potential overrepresentation of immune-based processes because of the Immunochip design bias^17^. As expected, P-Genes (384 EA, 253 AsA) were enriched in immunologically-driven functional categories and pathways; exclusion of P-Genes resulted in only minor alterations to overall categorization in either ancestral background (Supplemental Fig. S2).

### Delineation of signaling pathways identified by ancestry-specific SNP-associated genes

**Figure 3:**
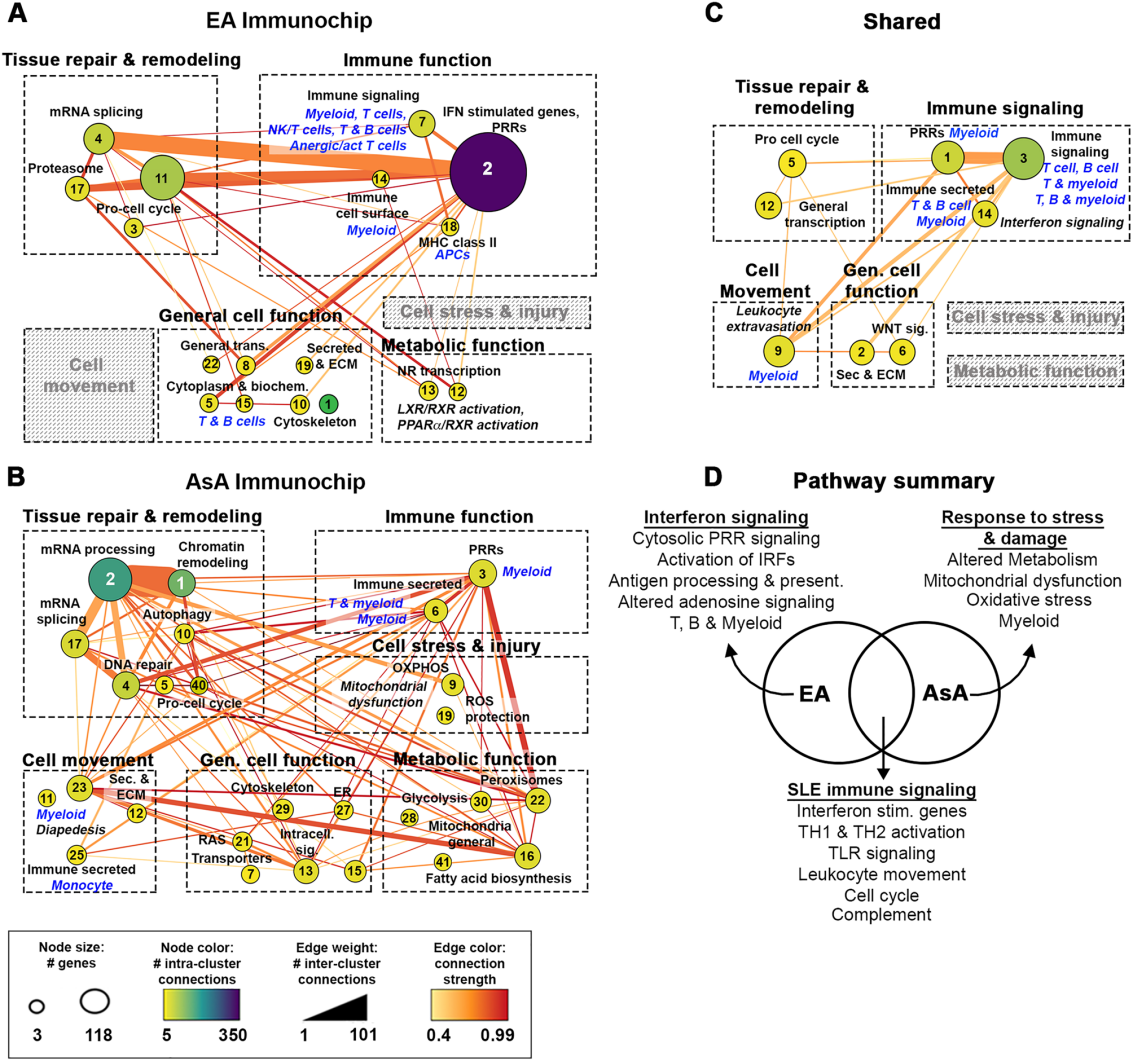
Key pathways determined by EA and AsA-associated genes. Cluster metastructures for EA (**A**), AsA (**B**) and the shared gene cohort (**C**) were generated based on PPI networks, clustered using MCODE and visualized in Cytoscape. Cluster size indicates the number of genes per cluster, edge weight indicates the number of inter-cluster connections and color indicates the number of intra-cluster connections. Enrichment for each cluster was determined by BIG-C and IPA; clusters were then grouped and categorized according to overall function (immune, tissue repair, metabolic, motility or general). Grey boxes indicate categories lacking relevant clusters. (**D**) Venn diagram showing the number of overlapping pathways motivated by EA or AsA predicted genes. Representative pathways are listed.

To assess ancestry-driven key signaling pathways in greater detail, ancestry-based protein–protein interaction (PPI) networks consisting of EA-associated, AsA-associated, or ancestry-independent genes were constructed using STRING, visualized in Cytoscape and clustered using MCODE (Supplemental Table S3). To provide an additional level of functional annotation, clusters contributing to overall immune function, tissue repair, mechanisms of cellular stress, cell motility, metabolic function or general cell function were grouped together. EA-associated genes were dominated by the functional category for interferon stimulated genes observed in cluster 2 (118 genes) (Fig. 3A), along with multiple canonical pathways related to the activation of pattern recognition receptors and downstream type I interferon signaling (Supplemental Table S4). Cluster 7 revealed additional enrichment in lymphocyte activation and differentiation, such as the *TH1 and TH2 activation pathway* that was also represented in the shared gene network, and cellular enrichment for cells of myeloid and/or lymphoid origin. Notably, the EA-associated network lacked evidence of cell motility and cell stress/injury, whereas metabolic function was represented by clusters 12 and 13 enriched in retinoid X receptor activation (*LXR/RXR activation*, *PPARα/RXRα activation*) involved in the regulation of lipid metabolism, inflammation, and cholesterol bile acid catabolism. Pathways associated with SLE in AsA were indicative of a diverse range of biological processes with protein metabolic functions dominating clusters 2 and 17 (Fig. 3B), whereas clusters 3 and 6 were enriched in multiple canonical pathways related to cytokine production and signaling (Supplemental Table S4). Interestingly, genes linked to SNPs associated in the AsA cohort did not include a unique interferon signature, but instead coalesced into multiple small clusters related to mitochondrial dysfunction (clusters 9 and 19) and metabolism, evident in clusters 16, 22 and 30. Additionally, AsA-associated gene clusters were enriched in chromatin remodeling found in cluster 1, along with evidence of cell motility (clusters 11, 12, 23 and 25). AsA cellular enrichment was dominated by monocytes and myeloid lineage cells.

Pathways exemplified by SLE-linked genes in both EA and AsA appear to be a blend of the pathways enriched within each ancestry. Common pathways included *Interferon signaling*, *TH1 and TH2 activation pathway, Complement system* and *Leukocyte extravasation* (Fig. 3C and Supplemental Table S5). Figure 3D depicts a selection of both the unique and overlapping canonical pathways motivated by the EA-associated and AsA-associated gene sets. We also carried out a parallel series of bioinformatics analyses to determine the biological function of the full array of EA (1676) and AsA (1756) SNP-predicted genes, including those associated with both ancestries (Supplemental Fig. S3 and Supplemental Table S5). As expected, shared genes were evenly distributed throughout each large network and subsequent connectivity mapping revealed the addition of several new clusters to both the EA and AsA networks. For example, the full EA network gained several clusters contributing to cell motility enriched in integrin signaling and granulocyte diapedesis (clusters 34 and 35), whereas the enlarged AsA network gained multiple clusters enriched in immune function (clusters 9, 12 and 31) and interferon signaling (cluster 3), as well as enrichment in a more diverse array of cell types, including T and, B cells, neutrophils and NK/T cells. Both networks acquired a small cluster enriched in the functional category for reactive oxygen species (ROS) protection (EA cluster 22, AsA cluster 24) driven by the glutathione peroxidases *GPX4*, an antioxidant enzyme involved in ferroptosis^18^, and *GPX3* an ROS scavenger. Overall, the addition of the shared gene cohort to each network highlights many common pathways and biological functions, but still revealed ancestry-driven differences. Taken together, unique pathways identified via EA analyses appear dominated by immune-related signaling as well as T, B and myeloid cells, whereas those in AsA analyses are dominated by biological processes related to altered metabolism and mitochondrial dysfunction.

### Validation of AsA-enriched molecular pathways using summary GWAS data

**Figure 4:**
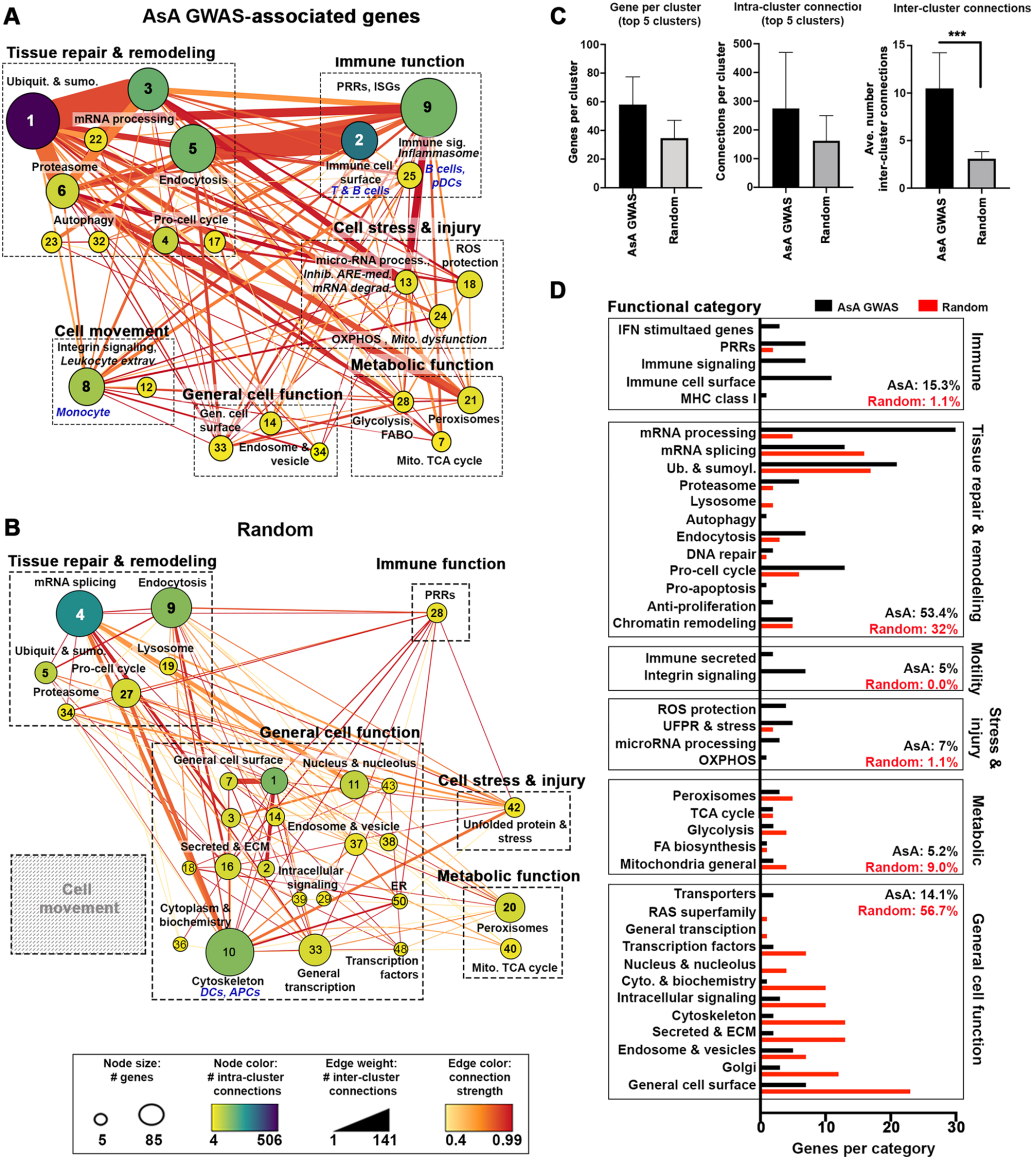
AsA Immunochip-based pathways are supported by summary GWAS from AsA SLE patients. Using SNP-predicted genes from the AsA GWAS validation SNP-set (**A**) or an equivalently sized cohort of random genes (**B**) metastructures were generated based on PPI networks, clustered using MCODE and visualized in Cytoscape. Cluster size indicates the number of genes per cluster, edge weight indicates the number of inter-cluster connections and color indicates the number of intra-cluster connections. Enrichment for each cluster was determined by BIG-C and IPA; clusters were then grouped and categorized according to overall function (immune, tissue repair, metabolic, motility or general). Grey boxes indicate categories lacking relevant clusters. (**C**) Quantitation of cluster size, intra-cluster connections and inter-cluster connections network is displayed. Error bars represent the 95% confidence interval; asterisks (***) indicate a p-value < 0.001 using Welch’s t-test. (**D**) Quantitation of AsA GWAS (black bars) and random (red) genes falling into each BIG-C category and grouped by overall functionality.

To test our pathway predictions, we combined summary data from previously reported GWA studies^7,9^ that identified 1350 SNPs associated with SLE in patients of East AsA ancestry (Supplemental Table S6). Of these SNPs, 68% were located in non-coding regions, 6.5% were in coding regions, 2.7% were in regulatory regions and 22% were located within or proximal to non-coding RNAs (Supplemental Fig. S4). Validation AsA-associated GWAS SNPs exhibited limited commonality when compared to Immunochip SNPs, with < 1% of either EA- or AsA-associated Immunochip SNPs overlapping GWAS SNPs, and only 3 SNPs common to all 3 datasets (Supplemental Fig. S4). We next applied our same bioinformatics-driven methodology to generate a validation gene cohort composed of 1321 E-Genes, 307 T-Genes, 17 C-Genes and 974 P-Genes (Supplemental Table S7). Connectivity mapping of all validation genes were used to create PPI networks that were clustered as described above (Fig. 4A). Examination of each cluster revealed functional similarity to those derived from AsA Immunochip-associated genes. For example, clusters 1, 3, 4, 5 and 6 share hallmarks of tissue repair and remodeling exemplified by categories for mRNA processing, pro-cell cycle and protein degradation (proteasome, lysosome, endocytosis). Additionally, we observed smaller clusters (21, 27 and 28) representative of processes involved in metabolic function, and clusters (13, 18 and 24) characteristic of cell stress and injury, including the *Inhibition of ARE-mediated degradation pathway* and *Mitochondrial dysfunction* canonical pathways (Supplemental Table S8). Cluster 9 contained a small interferon-stimulated gene signature consisting of *IFI27*, *IFI44* and *RSAD2* (Supplemental Table S3). Cellular categories were again dominated by monocytes, T cells, NK cells, B cells and plasmacytoid (p)DCs and are consistent with findings observed with AsA Immunochip-associated genes. In contrast to AsA-associated genes, where we observed large, highly connected clusters, an equivalent cohort of apparently random genes generally formed smaller clusters, exhibited fewer intra- and inter-cluster connections, and were primarily enriched in functional categories mostly related to basic cell function (general cell surface, secreted and ECM) (Fig. 4B,C). As shown in Fig. 4D, which displays the number of genes (and percentage of total genes) assigned to each functional category, random genes are skewed toward general cell function, whereas AsA-associated genes are more prevalent in the overall immune (15.3% of genes), tissue repair (53.4%) and cell stress (7%) categories. The random gene network also lacked evidence of cell movement and the diversity of cellular enrichment identified from AsA SNP-associated genes (Fig. 4B).

### Validation of AsA-enriched molecular pathways using gene expression data

**Figure 5:**
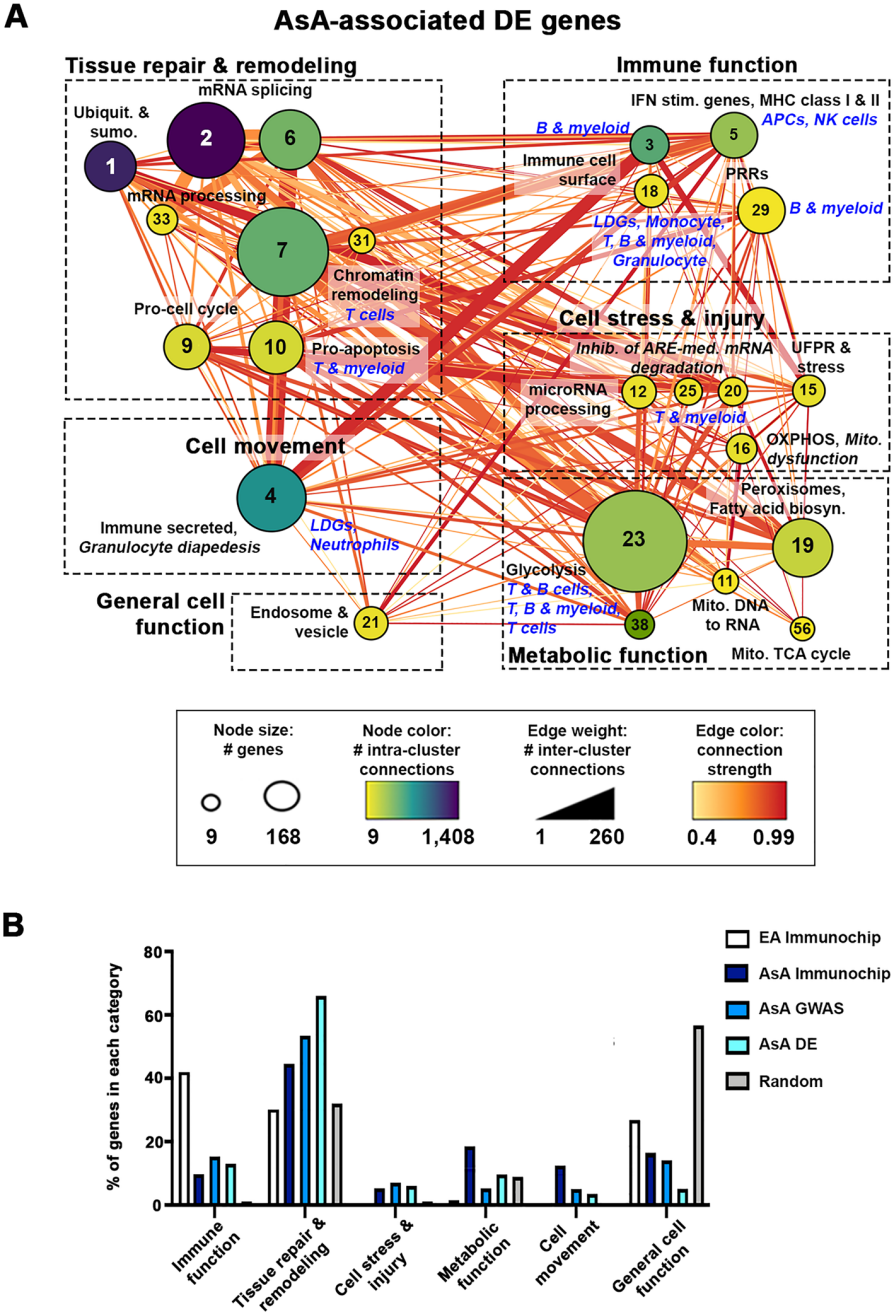
Asian-associated pathways are validated with gene expression data from AsA SLE patients. (**A**) Using differentially expressed (DE) genes from AsA whole blood samples (E-MTAB-11191), metastructures were generated based on PPI networks, clustered using MCODE and visualized in Cytoscape. Cluster size indicates the number of genes per cluster, edge weight indicates the number of inter-cluster connections and color indicates the number of intra-cluster connections. Enrichment for each cluster was determined by BIG-C and IPA; clusters were then grouped and categorized according to overall function (immune, tissue repair, metabolic, motility [Continued from previous page] or genera cell funtionl. (**B**) Bar graph showing the precent of associated (EA/AsA immunochip and AsA GWAS), differentially expressed and random genes falling into each overall functional category.

SNP-based pathway predictions were also tested in differential gene expression analyses from whole blood samples collected from AsA SLE patients with active disease and renal involvement compared with healthy controls (E-MTAB-11191, Supplementary Table S11). Overall, differential expression (DE) analysis revealed 5886 DE genes (DEGs) enriched in functional categories for interferon stimulated genes, gene expression, RNA processing and metabolism (Fig. 4 and Supplemental Fig. S5). A total of 685 AsA and 300 EA SNP-predicted genes were shared with AsA SLE DEGs, and 144 genes, representative of type I and type II interferon signaling, were shared among all three groups (Supplemental Fig. S6). Genes common to AsA DEGs and AsA SNP-predicted genes were enriched in RNA processing and translation, whereas DEGs shared with EA SNP-predicted genes were specifically enriched in type I interferon/cytokine signaling. Connectivity mapping and pathway analysis of AsA DEGs again revealed striking commonality with AsA SNP-associated genes from both the Immunochip and summary GWAS, exemplified by cluster enrichment in RNA splicing/processing, ubiquitylation, chromatin remodeling and metabolic function (glycolysis, TCA cycle, fatty acid biosynthesis and peroxisomes), and canonical pathways for *Spliceosome cycle* (cluster 2), *Inhibition of ARE-mediated RNA degradation* (clusters 12, 20 and 25), and *Mitochondrial dysfunction* (cluster 16) (Fig. 5A and Supplemental Table S9). The distribution of DE genes in AsA for each overall category were also similar to that observed with AsA-associated genes (Immunochip and GWAS) but differed from both the EA-Immunochip predicted or random genes (Fig. 5B).

**Figure 6:**
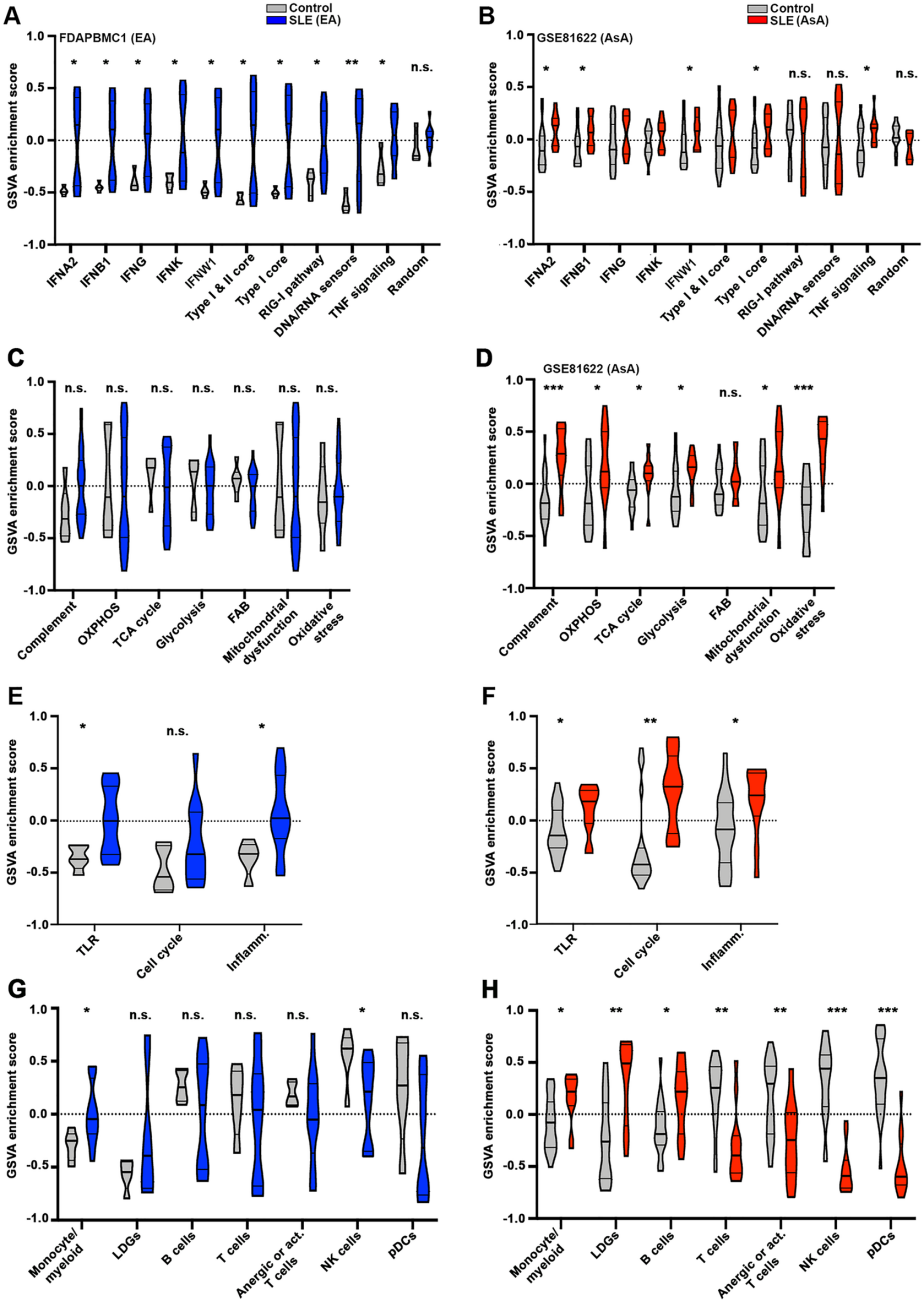
SNP-associated pathways inform gene signatures for GSVA analysis in patient PBMC datasets. GSVA enrichment scores were generated for PBMCs in EA and AsA SLE patients and healthy controls from FDAPBMC1 (EA-only patients and controls) and GSE81622 (AsA-only patients and controls). GSVA scores for type I and type II interferon-based gene signatures (**A**, **B**), metabolic gene signatures (**C**, **D**), cellular processes (**E**, **F**) and individual cell type signatures (**G**, **H**) are shown. Asterisks (*) indicate a p-value < 0.05 using Welch’s t-test comparing SLE to control.

To test the pathway predictions, Gene Set Variation Analysis (GSVA)^19^ was applied to determine the relative enrichment of gene signatures identified in peripheral blood mononuclear cell (PBMC) samples from SLE patients (EA and AsA) and controls (Supplemental Table S10). In FDAPBMC1, a dataset composed of EA patients (Supplemental Table S11), all 7 IFN gene signatures (IGS) and signatures for the RIG-I pathway and DNA/RNA sensors were strongly enriched in SLE PBMCs compared to controls (Fig. 6A). In contrast, only the signatures for IFNA2, IFNB1, IFNW1 and the Type I core were enriched in SLE PBMCs from AsA patients in GSE81622 (Fig. 6B). GSVA using a random group of genes did not separate SLE from controls in either dataset.

GSVA enrichment scores using signatures for complement activation and metabolic pathways, including mitochondrial oxidative phosphorylation (OXPHOS), the TCA cycle and glycolysis were able to separate AsA SLE patients, but not EA patients, from healthy controls (Fig. 6C,D). Consistent with our predictions, AsA SLE patients exhibited significant enrichment in signatures for mitochondrial dysfunction and oxidative stress. As shown in Fig. 6E–H, enrichment of a number of pathways and cell types in both EA and AsA SLE cohorts were noted, including TLR signaling, inflammasome, TNF signaling and monocyte/myeloid lineage cells, whereas AsA patients exhibited additional enrichment in pro-cell cycle, low density granulocytes (LDGs) and B cells. Varying degrees of T cell/NK cell lymphopenia were evident in both ancestral populations.

### Differential Inflammatory cell metabolism and activation status by ancestry

**Figure 7:**
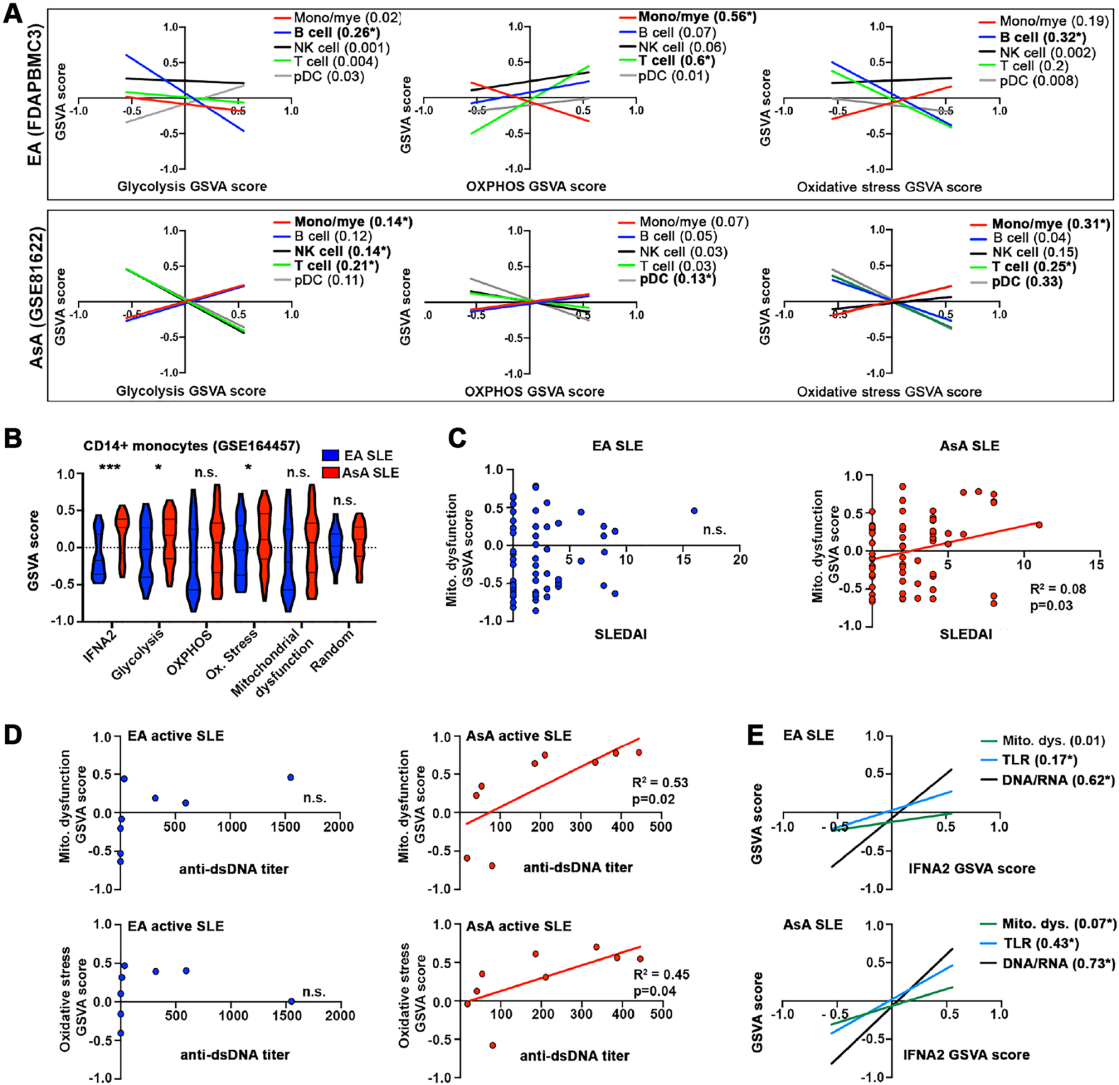
Linear regression to examine the relationship between cell types, biological processes and inflammatory cytokines. (**A**) Linear regression analysis showing the relationship between GSVA scores for glycolysis, oxidative phosphorylation or oxidative stress and individual cell types (pDCs, monocyte/myeloid, B cells, T cells and NK cells) for FDAPBMC1 (EA, upper panels) and GSE81622 (AsA, lower panels). In (**B**), GSVA enrichment scores for the indicated cellular processes were generated for purified CD14+ monocytes from EA and AsA SLE patients (GSE164457). Using GSE164457, linear regression was used to examine the relationship between cellular processes and SLEDAI (**C**), anti-dsDNA titers in active patients (SLEDAI ≥ 6) (**D**) and GSVA scores for IFNA2 (**E**). Categories with linear regression p values < 0.05 are in bold; R^2^ predictive values are listed after the GSVA enrichment category. *Asterisks indicate significant relationship between functional categories. N.s., not significant.

Because dysfunctional metabolic reprogramming can directly influence and exacerbate defective immune responses, we carried out linear regression analysis between the GSVA scores for individual cell signatures and the glycolysis and oxidative phosphorylation gene signatures to interrogate the metabolic status of immune cells from EA and AsA PBMC datasets. Enrichment scores for the glycolysis gene signature in AsA SLE patients exhibited positive correlation with both the monocyte/myeloid (R^2^ = 0.14, p = 0.03) and B cell signatures (R^2^ = 0.12, p = 0.051) (Fig. 7A). In contrast, glycolysis was not associated with gene signatures representing monocyte/myeloid cells, T cells or NK cells from EA patients, whereas the B cell category exhibited significant negative correlation (R^2^ = 0.26, p = 0.03). The gene signature for oxidative phosphorylation lacked positive correlation with all cell types in AsA patients, but did exhibit significant correlation (R^2^ = 0.6, p = 0.0003) with T cells in individuals of EA ancestry (Fig. 7A).

Since altered immune cell metabolism is associated with heightened oxidative cell stress responses in SLE^20^, we used the same linear regression approach to test the relationship between different immune cell types and GSVA enrichment scores for oxidative stress (Fig. 7A). Monocyte/myeloid cells from AsA patients were the only cell type demonstrating a significant positive relationship with the gene signature for oxidative stress (R^2^ = 0.3, p = 0.0008). These results were confirmed in purified CD14+ monocytes isolated from EA and AsA SLE patients (California Lupus Epidemiology Study, CLUES; GSE164457^21^) (Supplemental Table S11) showing significant enrichment of gene signatures for oxidative stress, glycolysis and IFNA2, along with a trend toward elevated OXPHOS and mitochondrial dysfunction, specifically in AsA SLE patients (Fig. 7B). Given that enhanced oxidative stress and concomitant mitochondrial dysfunction have been shown to correlate with disease activity, DNA damage, and autoantibody production^22–24^, we next examined the potential link between enrichment scores for mitochondrial dysfunction and SLE disease activity index (SLEDAI) score in these patients. GSVA scores for mitochondrial dysfunction demonstrated a significant positive relationship with SLEDAI in AsA but not EA SLE monocytes (Fig. 7C). Similarly, enrichment scores for oxidative stress and mitochondrial dysfunction showed a significant positive correlation with anti-dsDNA titers among AsA SLE patients with active disease (SLEDAI ≥ 6) compared to EA patients (Fig. 7D). In addition, overall complement C3 levels were lower in AsA patients and demonstrated a significant negative correlation with both anti-dsDNA and SLEDAI (Supplemental Fig. S7) in accordance with the observation that complement depletion and anti-dsDNA antibodies are often associated with elevated disease activity^25^.

Finally, given that mitochondrial dysfunction and nucleic acid sensing are potent inducers of interferons^26^, we tested for a relationship between IFNA2 enrichment scores and enrichment scores for the mitochondrial dysfunction, DNA/RNA sensors and TLR pathways (Fig. 7E). In EA patients, GSVA scores for the DNA/RNA sensors (R^2^ = 0.62, p < 0.0001) and TLR pathway (R^2^ = 0.17, p = 0.0013) signatures, but not mitochondrial dysfunction, were associated with IFNA2. In contrast, all three signatures exhibited a positive, significant correlation with IFNA2 in AsA patients.

## Discussion

SLE is a multisystem autoimmune disorder with a strong genetic contribution. The incidence of SLE varies widely across populations, with individuals of Asian, Hispanic and African ancestry demonstrating a three- to four-fold increase in disease prevalence compared to their European counterparts^27^. The advent of candidate gene, Immunochip and genome wide association studies (GWAS) has transformed our understanding of SLE genetics. However, it remains unclear how genetic ancestry contributes to the incidence, clinical heterogeneity and variation in disease outcomes among SLE patients. Specifically, AsA patients develop SLE at a younger age and with more severe manifestations, including lupus nephritis (LN)^28^. Whereas increased genetic risk burden in AsA individuals has been hypothesized to account for the increased prevalence of SLE in this population^9^, it does not provide adequate explanation for accelerated disease progression, variation in treatment response, or more extensive organ damage, especially with regard to the development of LN. Our observations suggest that, in addition to higher risk load, underlying differences at the genetic level may significantly influence the dominant biological pathways operative within each ancestry. Here, we show that by identifying a comprehensive list of genes implicated by GWAS and linking them to biologic pathways, we can provide a broader perspective on the genetic influences of SLE. It may also inform on health disparities particular to specific ancestries.

To accomplish this, we adopted a similar strategy to that recently described^11^, employing statistical and computational analyses along with data acquired from functional genomic assays to map the overall gene expression landscape of SLE and further define the disease-associated pathways. It is important to highlight that the SNPs examined here are generally present in both EA and AsA populations, but differences in allele frequencies suggest some SNP-predicted genes and pathways may be more influential in one ancestry over another. Rather than focusing on only a very limited number of top associations, this approach used a less stringent significance threshold to allow for the integration of more SNPs that could ultimately be pruned and mapped to a larger cohort of genes and pathways. Using SNPs discovered via Immunochip-based association studies, eQTL analysis identified 631 SNPs associated with 1955 E-Genes (730 EA, 1225 AsA). In the Asian ancestry studies, nearly 60% of SNPs were eQTLs, compared to 29% in EA. While eQTLs represented a higher proportion of SNPs associated with SLE in AsA across all genomic functional categories, it was of note that eQTLs linked to ncRNAs were nearly 3 times as frequent in AsA compared to EA patients. The disparity in distribution likely represents the heterogeneous genetic disposition uniquely affecting EA and AsA SLE patients. ncRNAs are a class of mRNA-like transcripts, typically > 200 nucleotides in length, that lack protein coding potential and serve as important regulators of gene expression by actions at the transcriptional, post-transcriptional and post-translational levels^29^. Several ncRNA eQTLs identified here were associated with anti-sense RNA E-Genes, including *IFNG-AS1* and *IL12A-AS1,* both of which are involved in the regulation of their cognate sense protein-coding genes^30,31^. Increasing evidence points to an important role for ncRNAs in the differentiation, polarization and activation of both myeloid and lymphoid lineage immune cell types^32^. Furthermore, abnormal ncRNA expression is associated with mitochondrial dysfunction-induced oxidative stress in a number of pathological conditions, including SLE^33–35^.

Overall, genes linked to SNPs associated with SLE in AsA cohorts were enriched in processes related to leukocyte migration, PRR signaling and RNA processing, and further detail provided by protein–protein interaction network and pathway analysis revealed multiple clusters enriched in translation/RNA processing, metabolic function, chromatin remodeling, cell stress and mitochondrial dysfunction. In contrast, these pathways, particularly mitochondrial dysfunction and cellular stress responses, were absent from the network analysis of EA SNP-associated genes. Instead, SLE-associated genes in EA data tended to be heavily influenced by immune processes, including the *Role of RIG-I in antiviral innate immunity, Antigen presentation,* and the *SLE in T cell signaling pathway*, as well as the functional category for interferon stimulated genes. Cellular enrichment categories were overwhelmingly dominated by T cells, B cells and myeloid cells, and is consistent with previous findings showing increased myeloid/monocyte gene signatures in EA ancestry independent of medication usage (i.e. SLE standard of care drugs including) and autoantibody production^36^. SNP-predicted pathways common to both EA and AsA were also enriched for myeloid and lymphoid lineage signaling (i.e. *TH1/TH2 activation signaling* and *IL12 signaling and production in macrophages),* along with the well described role of interferons in SLE.

To test our Immunochip SNP-predicted pathway-based findings, we turned to summary GWAS data from AsA SLE patients to create a second “validation” cohort of genes. While little numerical overlap between Immunochip SNPs and those derived from the AsA validation meta-analysis GWAS was observed, network-based analysis of SNP-predicted genes demonstrated a striking resemblance between pathways predicted by the Immunochip and those from the validation gene set, with multiple large clusters enriched in mRNA processing, degradation and nucleic acid sensing, along with smaller clusters enriched in metabolic activity and mitochondrial dysfunction. Conversely, network generation from an equivalently-sized random gene cohort contained smaller clusters, fewer intra- and inter cluster connections and exhibited little functional similarity with pathways predicted by AsA- or EA-associated genes. Nonetheless, consistent with our AsA Immunochip findings, SNPs within or proximal to ncRNAs were also highly prevalent in the validation dataset, accounting for 22% (299/1350) of total SNPs.

Recently, Wang and colleagues used ancestry-dependent and trans-ancestry meta-analyses to identify 38 novel non-HLA SLE loci^37^. Six disease variants were associated with SLE exclusively in East Asian populations of which 5 (*HIP1*, *TNFRSF13B*, *PRKCB*, *DSE* and *PLD4*) were linked to genes also identified here. Two of these genes are involved in antibody production, including *TNFRSF13B* that encodes the receptor for BAFF and plays a critical role in B cell development and survival, and *PRKCB,* a protein kinase C family member that regulates B cell activation via BCR-induced NF-κB activation^38^. These data suggest that different mechanisms may exist for antibody regulation between AsA and EA populations, whereby pathogenic antibody levels observed more frequently in non-Europeans may contribute to the higher prevalence of SLE in these populations. In fact, data presented here showing enrichment of B cell signatures and positive correlation between B cells and glycolysis may be indicative of increased B cell activation specifically in SLE patients of Asian ancestry. Nonetheless, by integrating all SLE SNP association-predicted genes into functional pathways, we have been able to detect additional differences contributed by ancestry such as those related to stress responses and metabolic dysfunction.

Pathway observations based on genetic findings were also tested in differential gene expression data. The biological pathways determined by AsA-associated DEGs from whole blood samples exhibited impressive similarity to those coupled to AsA SNP-associated genes and included evidence of metabolic and mitochondrial dysfunction, oxidative stress, cell death and gene expression/chromatin remodeling related processes. Furthermore, these results were validated via GSVA analysis in a second expression dataset from PBMCs showing that gene signatures for these processes were specifically enriched in samples from AsA, but not EA SLE patients. Despite the differences in EA and AsA revealed by network pathway analysis, SNP-predicted cellular enrichment common to both ancestral backgrounds included a wide variety of immune cell types, including APCs, T cells, B cell, myeloid, monocytes, LDGs and neutrophils. Additionally, while B cells and LDGs were enriched specifically in AsA SLE patients, the GSVA-based expression pattern demonstrating enrichment in monocyte/myeloid cells and reduced expression of T/NK and APCs was similar between EA and AsA SLE patients and is consistent with the involvement of these cell types in lupus pathogenesis.

Studies examining whether ancestry affects the clinical phenotype of SLE are complicated by the overwhelming heterogeneity of disease manifestations, especially with respect to organ involvement. Nonetheless, many genetic polymorphisms have been associated with specific phenotypes, autoantibody profiles, and/or clinical outcomes in SLE. For example, FcG receptor subtypes, such as FCGR3B have been significantly associated with LN and the presence of pathogenic autoantibodies, although it remains unclear whether there is a genetic basis for end-organ involvement based on ancestry^39^. FCGR3B is almost exclusively expressed by neutrophils and low copy number is associated with glomerulonephritis^39^. The SNP-predicted pathways described here suggest the presence of different biological mechanisms driving SLE. Importantly, we observed differential enrichment of these pathways in EA and AsA SLE data and thus these pathways may help explain some of the heterogeneity in SLE prevalence and severity across ancestral populations. This is not without precedent, as SNP-predicted genes from African Ancestry (AA) SLE patients identified pathways related to B cell signaling^11^ and these findings are consistent with reports showing that AA patients experience increased B cell activation and plasma cells compared to their EA counterparts^36,40,41^. Dominant pathways identified by AsA-associated SNPs and verified by gene expression data include those linked to mitochondrial and/or metabolic dysfunction and subsequent oxidative injury. Mitochondrial components can directly stimulate immune receptors by acting as damage-associated molecular patterns (DAMPs)^42^. They also function as an important source for reactive oxygen species (ROS), as well as facilitate inflammasome activation^42^. In SLE patients, defective mitochondrial function can increase oxidative stresses characterized by increased lipid peroxidation, elevated ROS production and decreased levels of antioxidant enzymes, such as superoxide dismutase (SOD), catalase (CAT) and glutathione peroxidase (GPx)^22^. In fact, enhanced mitochondrial ROS synthesis in SLE has been shown to directly contribute to type I IFN responses^43^, findings that are consistent with our results showing a significant correlation between the gene signatures for mitochondrial dysfunction and IFNA2 driven by the AsA analyses.

Importantly, mitochondrial dysfunction and associated oxidative damage has also been shown to be a leading factor in the development of chronic and acute renal diseases^44^. Lupus nephritis (LN) is a major risk factor for overall morbidity and mortality in SLE. Previous work has shown that SLE patients of Asian descent are at significantly higher risk for the development of LN^45^, whereas European genetic ancestry was found to be protective against renal disease^46^. In accordance with these findings, enrichment scores for mitochondrial dysfunction and oxidative stress significantly correlated with anti-dsDNA titers in AsA SLE patients with active disease compared to EA patients. These observations are intriguing in light of recent findings by Tsai and colleagues indicating cross-talk between oxidative stress and ncRNAs can trigger autoimmune reactions and perpetuate tissue damage in SLE patients^33^. Although the reason for the disparity in renal involvement between ancestral backgrounds has not been fully delineated, we could posit that differential expression of ncRNAs in kidney tissues may contribute to the significant immune dysregulation affecting Asian SLE patients. Whether this might be related to expression of specific regulatory ncRNAs or is a consequence of overall ncRNA burden will require further examination. However, in support of the former, several groups have reported aberrant cell type specific activation linked to the altered expression of non-coding microRNAs, including miR-31, miR145 and miR224 involved in T cell activation, that may be participating in LN pathophysiology^47^. Although these and other studies examining ncRNAs were conducted using samples from patients primarily of Asian ancestry, their results do not obviate the possibility that similar effects occur in EA SLE patients. This highlights a major limitation of the current study: there is no direct evidence that Asian patients experience a higher degree of mitochondrial dysfunction and oxidative stress-induced tissue damage compared to other ancestral backgrounds. As such, the relationship between altered ncRNA expression, immune dysfunction and tissue injury remains unclear. Nonetheless, genetic and gene expression evidence provided here suggests that AsA ancestral populations may be predisposed to altered cell stress responses where excessive mitochondrial oxidative stress derived ROS and RNS may trigger autoimmune reactivity, increasing cell death and promoting destructive inflammatory reactions in susceptible individuals.

Our genetic and gene expression analyses suggest that metabolic dysfunction is a key feature more prevalent in individuals of Asian compared to European ancestry. Reprogramming of immune cell metabolism is required to sustain the energy demands of effector functions, such as differentiation, clonal expansion, secretion of proinflammatory mediators, phagocytosis, and chemotaxis^48^. Metabolic dysfunction is common in kidney disease and recent work by our group has demonstrated that altered metabolic function in lupus-affected tissues (kidneys and skin) reflect damage induced by myeloid cell infiltration^16^. In myeloid lineage cells (monocyte/macrophages), enhanced glucose metabolism, either via glycolysis (characteristic of M1 macrophages) or OXPHOS (characteristic of M2 macrophages) is essential for cell survival, proliferation and to sustain various effector responses^49^. Regression analysis using PBMC and purified CD14+ monocytes isolated from SLE patients revealed a significant positive correlation between monocyte signatures from AsA subjects and glycolysis, but not OXPHOS, suggesting they are likely to be metabolically M1 in nature. Glycolysis was also correlated with B cells in AsA individuals suggesting that B cells, along with monocyte/myeloid cells in this patient population, maintain an activated phenotype.

A limitation of the current study is that the computational and experimental approaches are inferential. By attempting to provide a comprehensive translation of all GWAS findings, it remains challenging to determine those genes that are causal and those that may be considered false-positive associations. To address this, PPI networks and unsupervised MCODE clustering based on interaction strength help exclude those genes lacking strong connections within or between similarly functioning clusters to uncover biologically-relevant pathways. In comparison to networks generated from random SNPs and genes, the densely connected PPIs and highly significant gene-set annotation enrichments displayed in the current manuscript are clearly distinct from background noise and often implicate unexpected molecular pathways that may be ancestrally-motivated.

Other limitations of this study include those related to the data integrated in our pipeline. Initial genetic findings were based on the Immunochip which was specifically constructed for deep replication of major autoimmune and inflammatory diseases and fine-mapping of established GWAS loci^17^. As this platform was designed for use in European populations, it may have introduced considerable bias, especially with respect to the dominance of immune and interferon-mediated signaling pathways observed in our EA analyses. Given that type I IFN is a primary pathogenic factor in SLE regardless of ancestral background, additional analyses, including an examination of pathways predicted from summary AsA GWAS, differential gene expression from AsA whole-blood samples and GSVA enrichment of interferon gene signatures in AsA patients confirmed the well-established association between SLE and IFN across ancestral populations. Nonetheless, the specific triggers of IFN production and the mechanisms by which interferon signaling perpetuate the cycle of autoreactive cells and autoreactive antibody production are not completely clear, and evidence presented here suggest that different mechanisms exist for autoantibody and IFN regulation between EA and AsA populations. Finally, the ability to map SNPs to implicated genes is limited to known SNP-to-gene relationships included in Ensembl’s variant effect predictor (VEP), Genotype-Tissue Expression (GTEx) and Human ACtive Enhacer to interpret Regulatory variants (HACER) databases. However, of all SNP-predicted genes, only those encoding proteins included on the STRINGdb platform were incorporated into PPI interaction networks. While this is useful for filtering out unrelated genes, it excludes the large number of non-coding genes implicated in our SNP-to-gene predictions, an important consideration given the growing evidence highlighting the contributions of long non-coding RNAs and microRNAs in SLE^34,47^. Similarly, the ability to annotate gene clusters functionally is limited and potentially biased by the data underlying the numerous enrichment platforms used in our pathway analyses. It is for this reason that multiple platforms were used, including Ingenuity Pathway Analysis (IPA), EnrichR, which pools a myriad of public databases, and cell (IScope) and functional (BIG-C) analytic tools, to obtain orthogonal and reproducible annotations.

In conclusion, the SNP-associated predicted genes and gene expression profiles outlined here implicate fundamental differences in lupus molecular pathways enriched in EA and AsA ancestral populations. Our findings suggest that while certain pathways may be enriched in one ancestral population over another, it is important to note that those pathways may not be active within every patient of a given ancestry and may be active in a patient from different ancestry. Systems bioinformatics and assessment using gene signature enrichment analyses revealed alterations in cellular metabolism and cell stress signatures that may be more prevalent in patients of Asian ancestry. Whereas treatment strategies aimed at restoration of metabolic and/or antioxidant pathways are not straightforward, the current findings suggest that targeting metabolic dysfunction may hold promise for AsA patients who respond poorly to conventional therapies. Indeed, mTOR pathway modulators such as N-acetyl cysteine and rapamycin appear to be viable therapies for reducing disease activity^50,51^. Recently, pioglitazone, a peroxisome proliferator-activated receptor (PPARg) agonist, was found to ameliorate nephritis symptoms in lupus-prone animals^52^. Together, our study demonstrates that multilevel analysis is capable of defining gene regulation that not only reflects differences in EA and AsA populations, but also represents candidate pathways that may be the target of ancestry-specific therapies.

## Material and methods

### Genomic functional categories

The Variant Effect Predictor (VEP) tool available on the Ensembl genome browser 93 was used for annotation information to specify SNPs located within non-coding regions, including micro (mi)RNAs, long non-coding (lnc)RNAs, splice region variants, non-coding transcript exon variants, introns and intergenic regions. Regulatory regions include transcription factor binding sites (TFBS), promoters, enhancers, repressors, promoter flanking regions (PFRs) and open chromatin (OCRs). Coding regions were broken down further and include 5’UTRs, 3’UTRs, synonymous and nonsynonymous (missense and nonsense) mutations. The online resource tool HaploReg (version 4.1^53^; was also used to identify DNA features, regulatory elements and assess regulatory potential.

### Identification of SLE-associated SNPs and predicted genes

SLE Immunochip studies identified single nucleotide polymorphisms (SNPs) significantly associated with SLE in EA (6748 cases; 11,516 controls, p < 1 × 10^−6^)^8^. Because of the lower power of the East Asian Immunochip analysis reported in Sun et al.^6^ (2485 cases and 3947 controls from Koreans (KR), Han Chinese (HC) and Malaysian Chinese (MC)), we identified 700 SNPs from 578 associated regions using a significance threshold of p < 5 × 10^−3^). Because of the extensive linkage disequilibrium in the HLA region, SNPs in the region spanning chr6:28014374-33683352 were omitted from the analysis. Asian validation SNPs were previously described^7,9^. Expression quantitative trait loci (eQTLs) were then identified using GTEx version 8 (GTEXportal.org^54^) and the Blood eQTL browser database^13^ and mapped to their associated eQTL expression genes (E-Genes). To find SNPs in enhancers and promoters, especially in intergenic regions, and their associated transcription factors and downstream target genes (T-Genes), we queried the atlas of Human Active Enhancers to interpret Regulatory variants (HACER)^15^ and the GeneHancer database^14^. To find structural SNPs in protein-coding genes (C-Genes), we queried the human Ensembl genome browser (GRCh38.p12) and dbSNP. Several additional databases were used to generate loss-of-function prediction scores, including SIFT4G^55,56^ and PolyPhen-2^57^. All other SNPs were linked to the most proximal gene (P-Gene) or gene region as previously detailed^8^. Predicted genes were examined as equal entities; no gene, regardless of provenance, was given more weight or importance over another type. For overlap studies, Venn diagrams were computed and visualized using InteractiVenn^58^. All predicted genes were divided into an EA, AsA or shared group depending on the ancestral designation of the original SLE-associated SNP. The random gene cohort was generated using the Random Gene Set Generator.

### Differential gene expression analysis

For DEG analysis of E-MTAB-11191, affymetrix probes were annotated with custom BrainArray (BA) chip definition files (CDFs)^59^ as previously described^60^. Any probes with different Affymetrix and BA gene annotations were excluded. GCRMA-normalized expression values were variance corrected using local empirical Bayesian shrinkage before calculation of differentially expressed genes (DEGs) using the ebayes function in the BioConductor LIMMA package. P-values were adjusted for multiple hypothesis testing using the Benjamini–Hochberg False discovery rate (FDR). Significant Affymetrix and BA probes within each study were merged and filtered to retain probes with a pre-set FDR < 0.2 which were considered statistically significant. This FDR threshold was employed to avoid falsely excluding genes of interest. This list was further filtered to retain only the most significant probe per gene in order to remove duplicate genes.

### Functional gene set analysis

For both ancestral groups, predicted gene lists were examined using Biologically Informed Gene Clustering (BIG-C; version 4.4.). BIG-C is a custom functional clustering tool developed to annotate the biological meaning of large lists of genes. Genes are sorted into 54 categories based on their most likely biological function and/or cellular localization based on information from multiple online tools and databases including UniProtKB/Swiss-Prot, gene ontology (GO) Terms, MGI database, KEGG pathways^61^, NCBI, PubMed, and the Interactome, and has been previously described^62,63^. I-Scope is a custom clustering tool used to identify immune infiltrates in large gene datasets, and has been described previously^64^. Briefly, I-Scope was created through an iterative search of more than 17,000 genes identified in more than 50 microarray datasets. These genes were researched for immune cell specific expression in 30 hematopoietic sub-categories: T cells, regulatory T cells, activated T cells, anergic T cells, CD4 T cells, CD8 T cells, gamma-delta T cells, NK/NKT cells, T & B cells, B cells, activated B cells, T, B & myeloid, monocytes, monocytes & B cells, MHC Class II expressing cells, monocyte dendritic cells, dendritic cells, plasmacytoid dendritic cells, Langerhans cells, myeloid cells, plasma cells, erythrocytes, neutrophils, low density granulocytes, granulocytes, platelets, and all hematopoietic stem cells. Enrichment of GO Biological Processes (BP) using Enrichr^65^ and the Ingenuity Pathway Analysis (IPA) platform provided additional genetic pathway identification.

### Network analysis and visualization

For SNP-predicted genes, only those encoding proteins included on the STRINGdb (version 1.3.2) platform were incorporated into protein–protein interaction (PPI) networks. To provide a quality control checkpoint, the significance of network connectivity was assessed, and we only moved forward with pathway analysis if the PPI enrichment p-value was < 1 × 10^−16^ (lowest p-value, highest possible probability estimate). Visualization of protein–protein interaction and relationships between genes within datasets was done using Cytoscape (version 3.6.1) software. Briefly, STRINGdb generated networks were imported into Cytoscape and partitioned with MCODE via the clusterMaker2 (version 1.2.1) plugin.

### Gene set variation analysis (GSVA)

The GSVA (V1.25.0) software package for R/Bioconductor and has been described previously. Briefly, GSVA is a nonparametric, unsupervised method for estimating the variation of pre-defined gene sets in patient and control samples of microarray expression datasets. The input for the GSVA algorithm was a gene expression matrix of log2 microarray of expression values and a collection of pre-defined gene signatures. Enrichment scores (GSVA scores) were calculated non-parametrically using a Kolmogorov-Smirnoff (KS)-like random walk statistic and a negative value for each gene set. GSVA gene signatures using official gene symbols are listed in Table S7). All interferon and cytokine signatures (core IFN, IFNB1, IFNA2, IFNW, IFNG and TNF) have been described previously. Metabolic signatures were based on literature mining and established IPA canonical pathways. Enrichment of each signature was examined in EA and AsA SLE patients and healthy control PBMCs from FDAPBMC1 for EA or GSE81622 for AsA. Differences between controls and SLE patient GSVA enrichment scores were determined using the Welch’s t-test for unequal variances in Graphpad PRISM 8.0.

### Regression models

For all linear models, GSVA scores for cell type and/or pathway in each sample were used as input. Simple linear regression was performed in Graphpad PRISM 8.0.

## Supplementary Information

Supplementary Tables.

Supplementary Figures.

## Data Availability

All microarray and RNA-seq datasets listed in this publication (GSE164457, GSE81622, FDAPBMC1 and EMTAB11191) are publicly available on the NCBI's database Gene Expression Omnibus (GEO) (https://www.ncbi.nlm.nih.gov/geo/).
