# Supplementary material for: Molecular pathways identified from single nucleotide polymorphisms demonstrate mechanistic differences in systemic lupus erythematosus patients of Asian and European ancestry
Source: Sci Rep. 2023 Apr 1;13:5339. doi: 10.1038/s41598-023-32569-6 (PMC10067935; doi:10.1038/s41598-023-32569-6)
Supplement: Supplementary file 2 — Supplementary Figures. [file 41598_2023_32569_MOESM2_ESM.pdf]

Supplementary Information

Molecular Pathways Identified from Single Nucleotide Polymorphisms Demonstrate Mechanistic Differences in Systemic Lupus Erythematosus Patients of Asian and European Ancestry

Katherine A. Owen, Kristy A. Bell, Andrew Price, Prathyusha Bachali, Hannah Ainsworth, Miranda C. Marion, Timothy D. Howard, Carl D. Langefeld, Nan Shen, Jinoos Yazdany, Maria Dall'era, Amrie C. Grammer and Peter E. Lipsky

Supplemental Figures and Legends

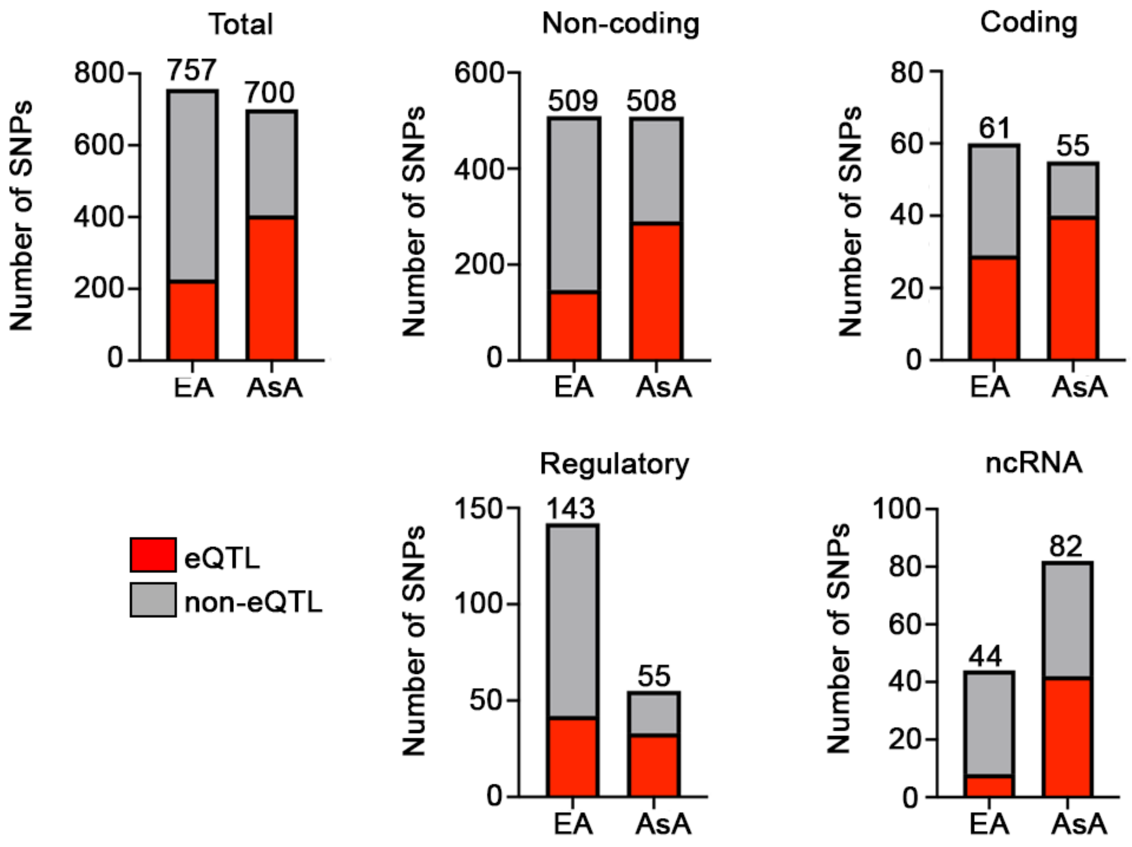

Figure S1. Immunochip SNPs exhibiting eQTL effects are more frequent in Asian Ancestry. EA and AsA Immunochip SNPs designated as eQTL via the GTEx and Blood eQTL browser databases were distributed into their genomic functional categories. Numbers above each bar indicate the total number of SNPs in each category. Red, eQTL; gray, non-eQTL.

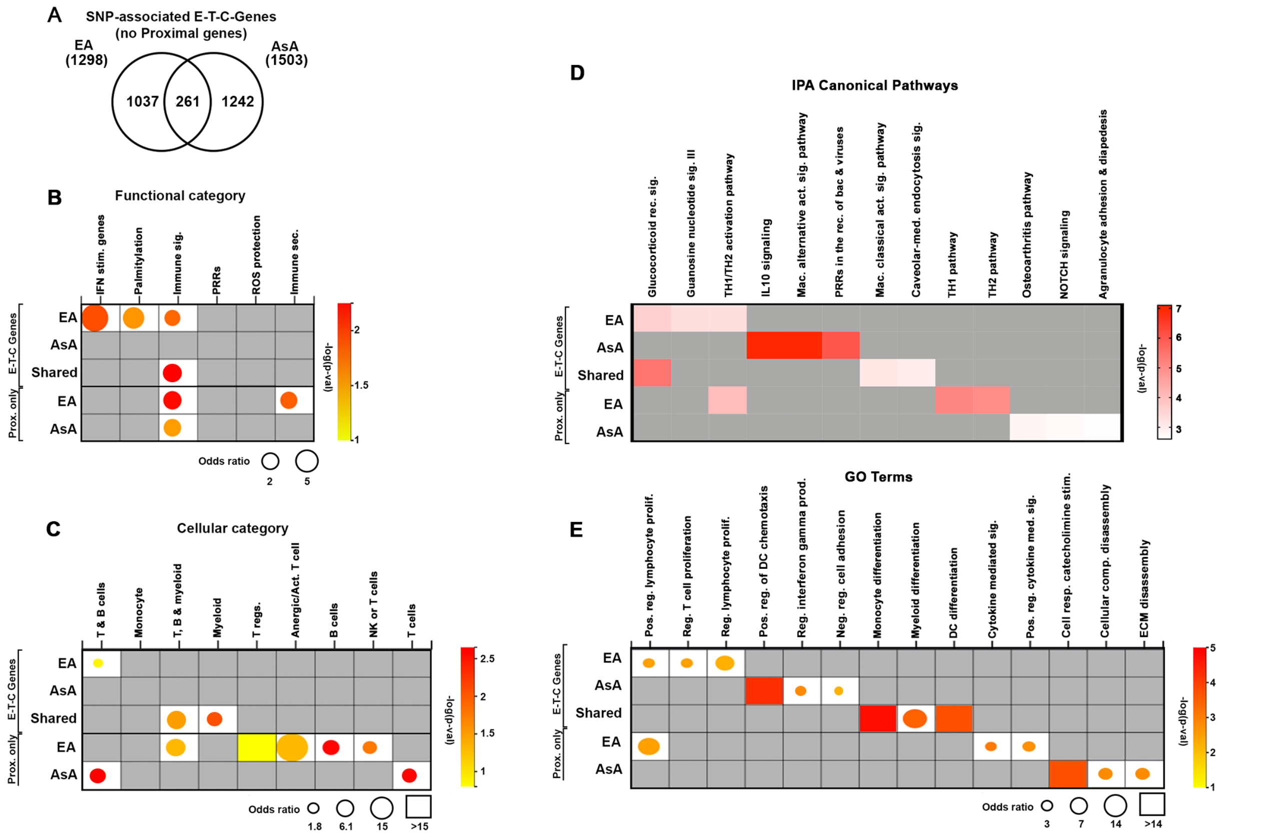

Figure S2. Functional characterization of SNP-associated E-T-C-Genes. (A) Venn diagram depicting the overlap between SNP associated E-T-C EA- and AsA genes (excluding P-Genes). (**B-C**) Bubble plots depict E-T-C ancestry-dependent and independent SNP-associated genes analyzed to determine enrichment using functional definitions from the BIG-C (Biologically Informed Gene Clustering) annotation library and I-Scope for hematopoietic cell enrichment. Enrichment was defined as any category with an odds ratio (OR) >1 and a –log (p-value) >1.33. EA and AsA P-Genes were analyzed separately. (**D**) Heatmap visualization of the top three significant IPA canonical pathways and (**E**) bubble plot showing gene ontogeny (GO) terms for each gene list organized by ancestry. Top pathways with OR >1 and –log (p-value) >1.33 are listed.

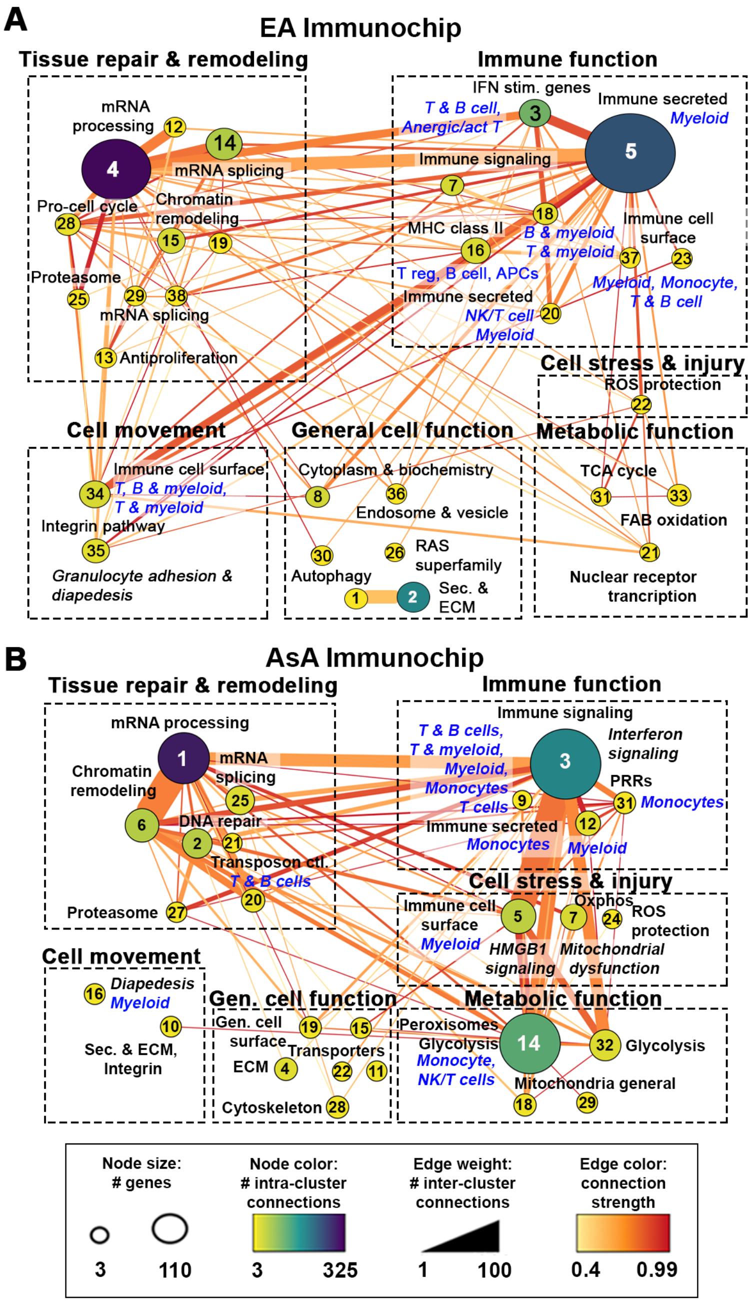

Figure S3. Key pathways determined by all EA and AsA-associated genes. Cluster metastructures using the full cohort of EA (**A**) and AsA (**B**) genes were generated based on PPI networks, clustered using MCODE and visualized in Cytoscape. Cluster size indicates the number of genes per cluster, edge weight indicates the number of inter-cluster connections and color indicates the number of intra-cluster connections. Enrichment for each cluster was determined by BIG-C and IPA; clusters were then grouped and categorized according to overall function (immune, tissue repair, metabolic, motility or general). Grey boxes indicate categories lacking relevant clusters.

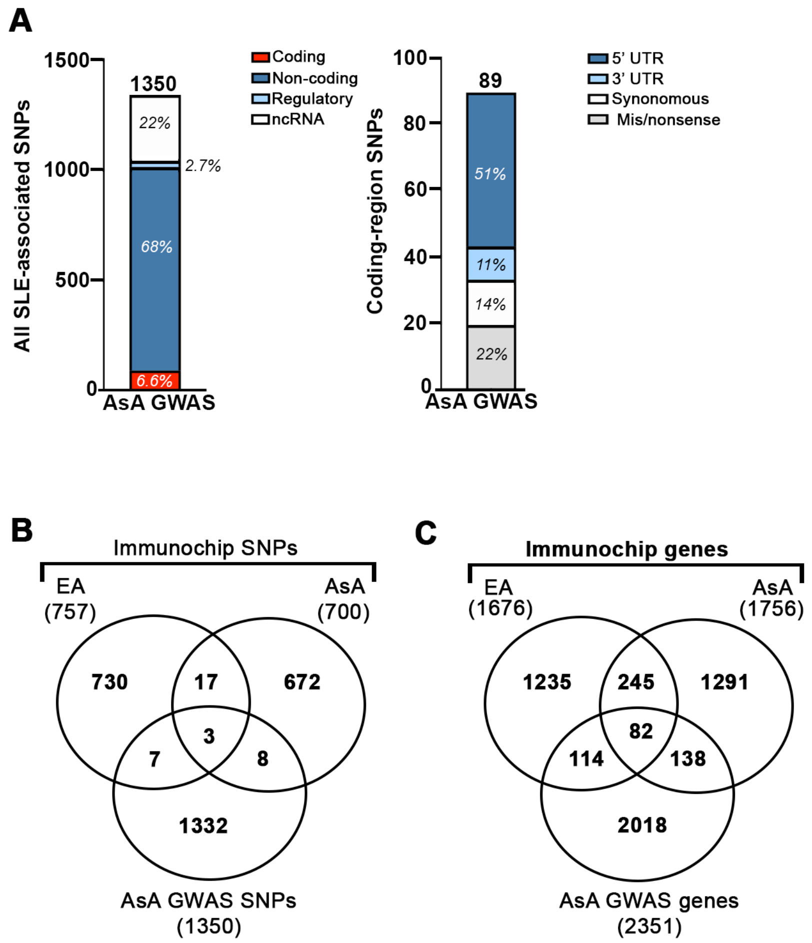

Figure S4. Distribution of genomic functional categories for GWAS validation cohort SNPs. (**A**) The genomic functional categories for all GWAS validation SLE SNPs was determined. Coding region SNPs were further broken down based on their location. Numbers above each bar indicate the total number of SNPs in each category. (**B-C**) Venn diagrams depicting the ancestral overlap of all Immunochip and GWAS SNPs and predicted genes.

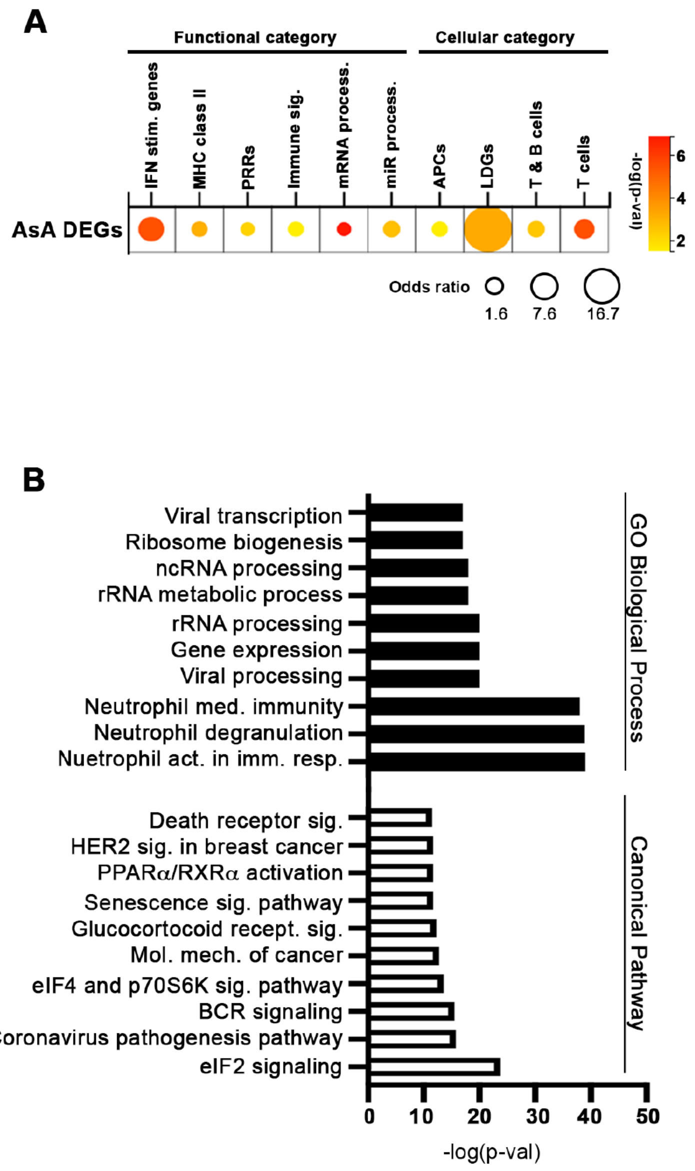

Figure S5. Key pathways determined by AsA differentially expressed genes. (**A**) Differentially expressed AsA genes were examined for functional and cellular enrichment using BIG-C and I-Scope, respectively. Bubble plot depicts significantly enriched categories (-log(p-value) > 1.33; OR > 1). (**B**) Top GO Biological and IPA canonical pathways (-log(p-value) > 1.33) for all AsA DEGs.

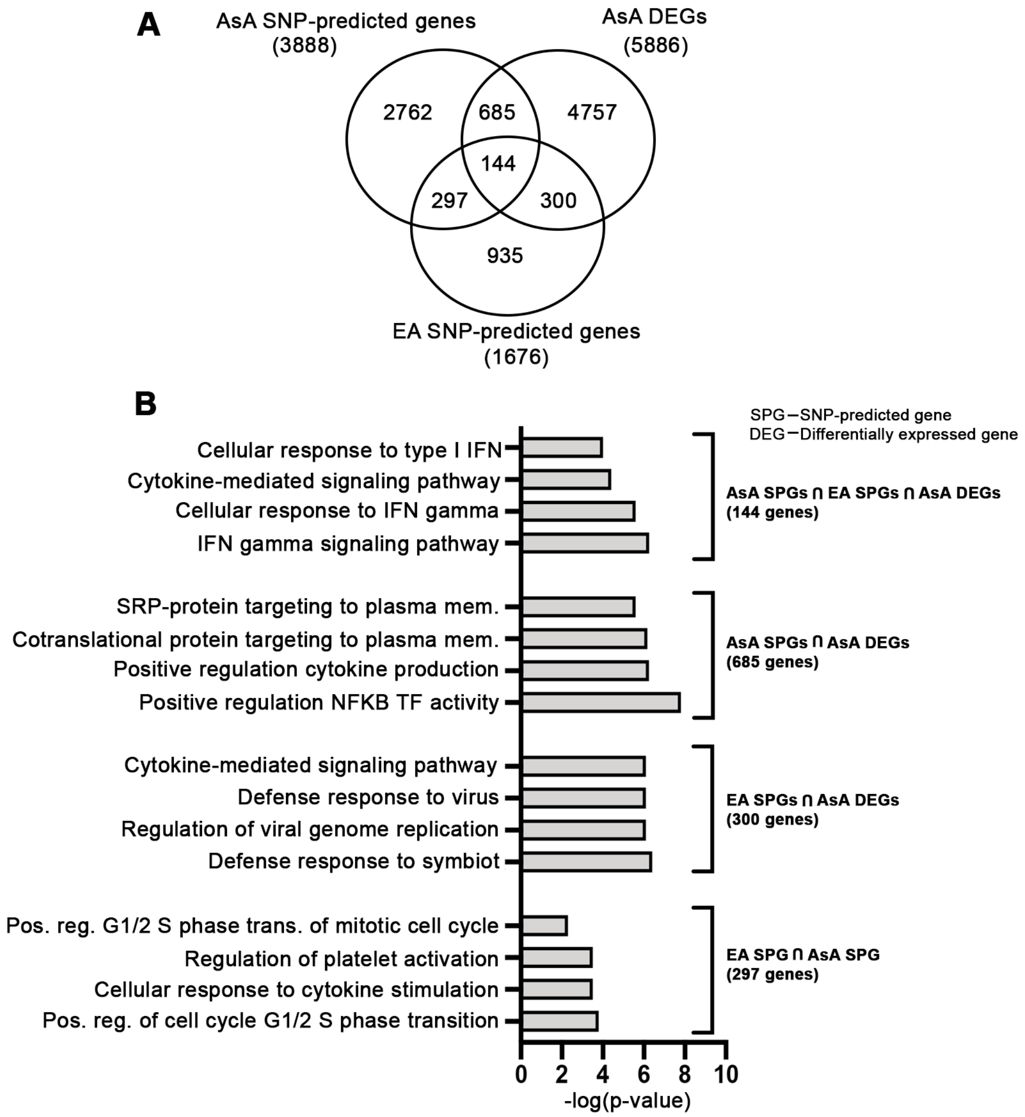

Figure S6. Key overlapping pathways determined by SNP-predicted and differentially expressed genes. (**A**) Venn diagram depicting the numerical overlap between AsA SNP-predicted genes (SPGs), EA SPGs and AsA DEGs. (**B**) Top GO Biological pathways determined by each group of overlapping genes (-log(p-value) > 1.33).

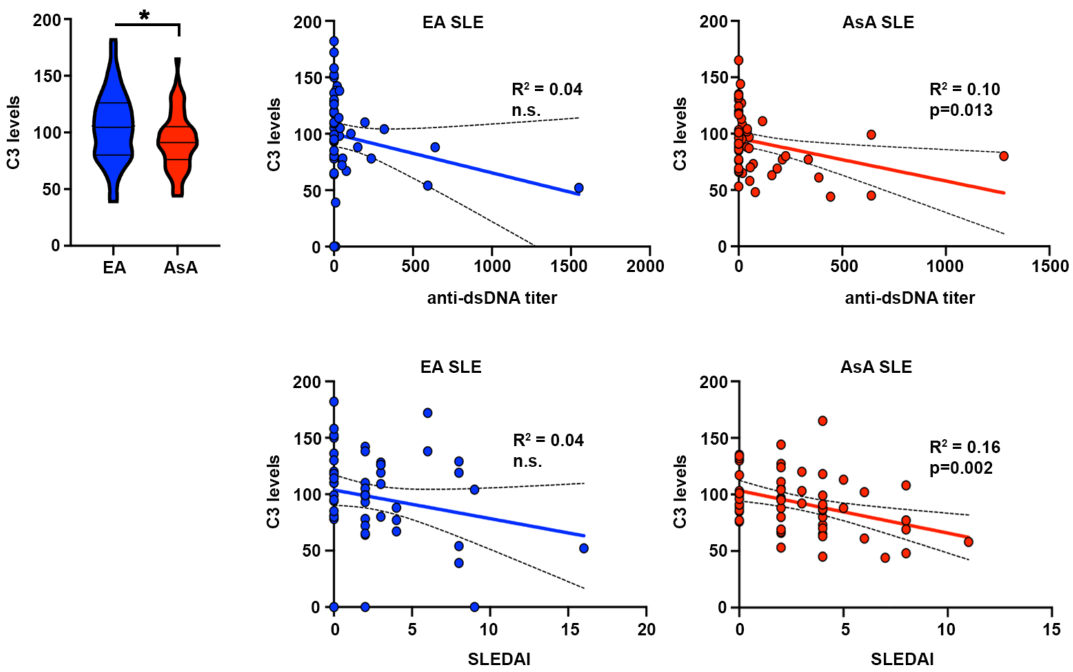

Figure S7. Complement depletion is associated with anti-dsDNA titers and SLEDAI in AsA SLE patients. (**A**) Comparison of complement C3 levels in EA and AsA SLE patients (GSE164457). Asterisks (*) indicate a p-value <0.05 using Welch's t-test. (**B-C**) Linear regression demonstrating the relationship between complement C3 levels and anti-dsDNA titers and disease activity as measured by SLE disease activity index (SLEDAI). R^2 predictive values and p-values are listed. N.s., not significant.
